# Supplementary material for: Evidence of Clinical Pathology Abnormalities in People with Myalgic Encephalomyelitis/Chronic Fatigue Syndrome (ME/CFS) from an Analytic Cross-Sectional Study
Source: Diagnostics (Basel). 2019 Apr 10;9(2):41. doi: 10.3390/diagnostics9020041 (PMC6627354; doi:10.3390/diagnostics9020041)
Supplement: Supplementary file 1 [file diagnostics-09-00041-s001.pdf]

Supplementary

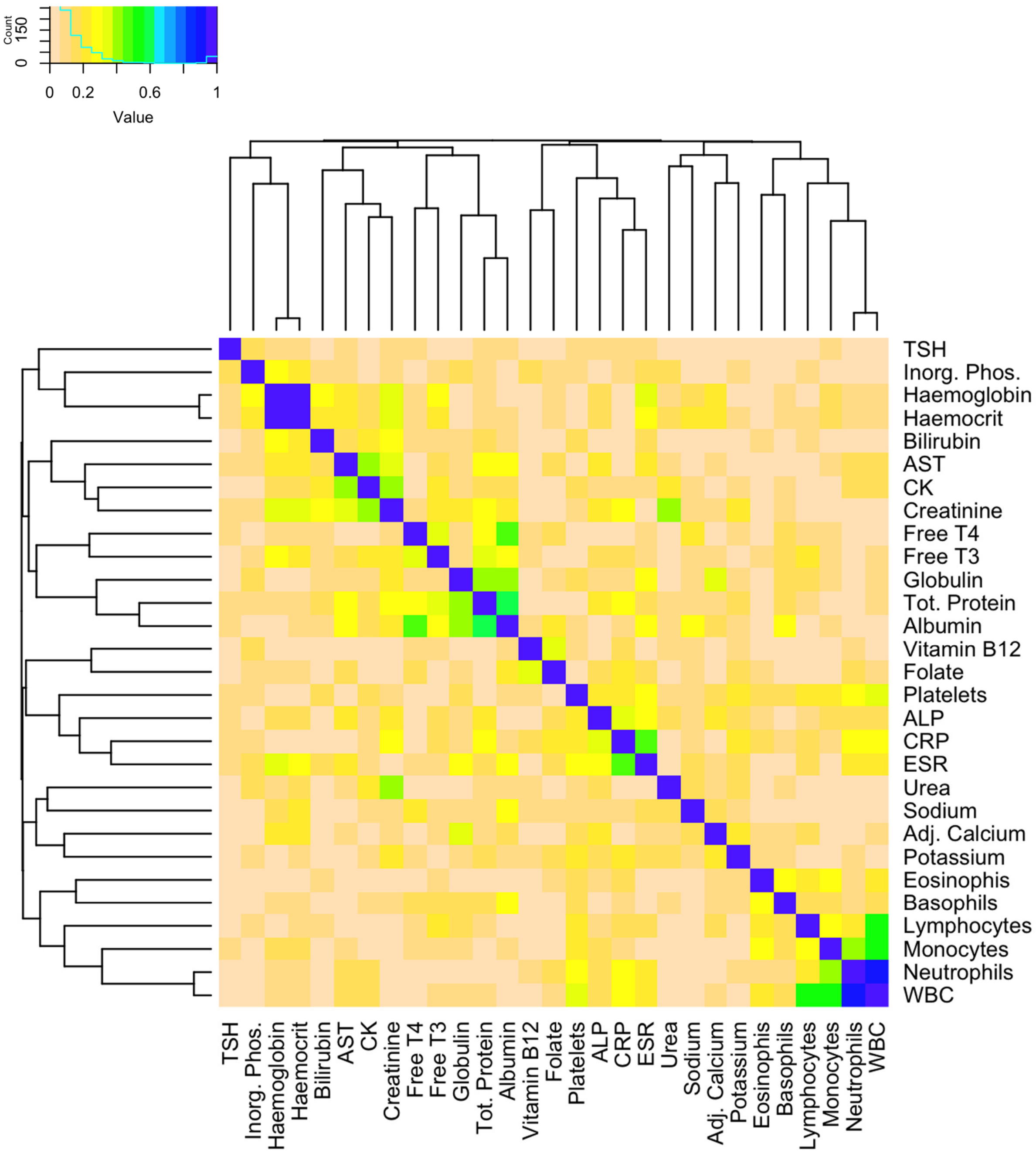

Figure S1. (Absolute) Spearman's correlations between laboratory tests across all samples.

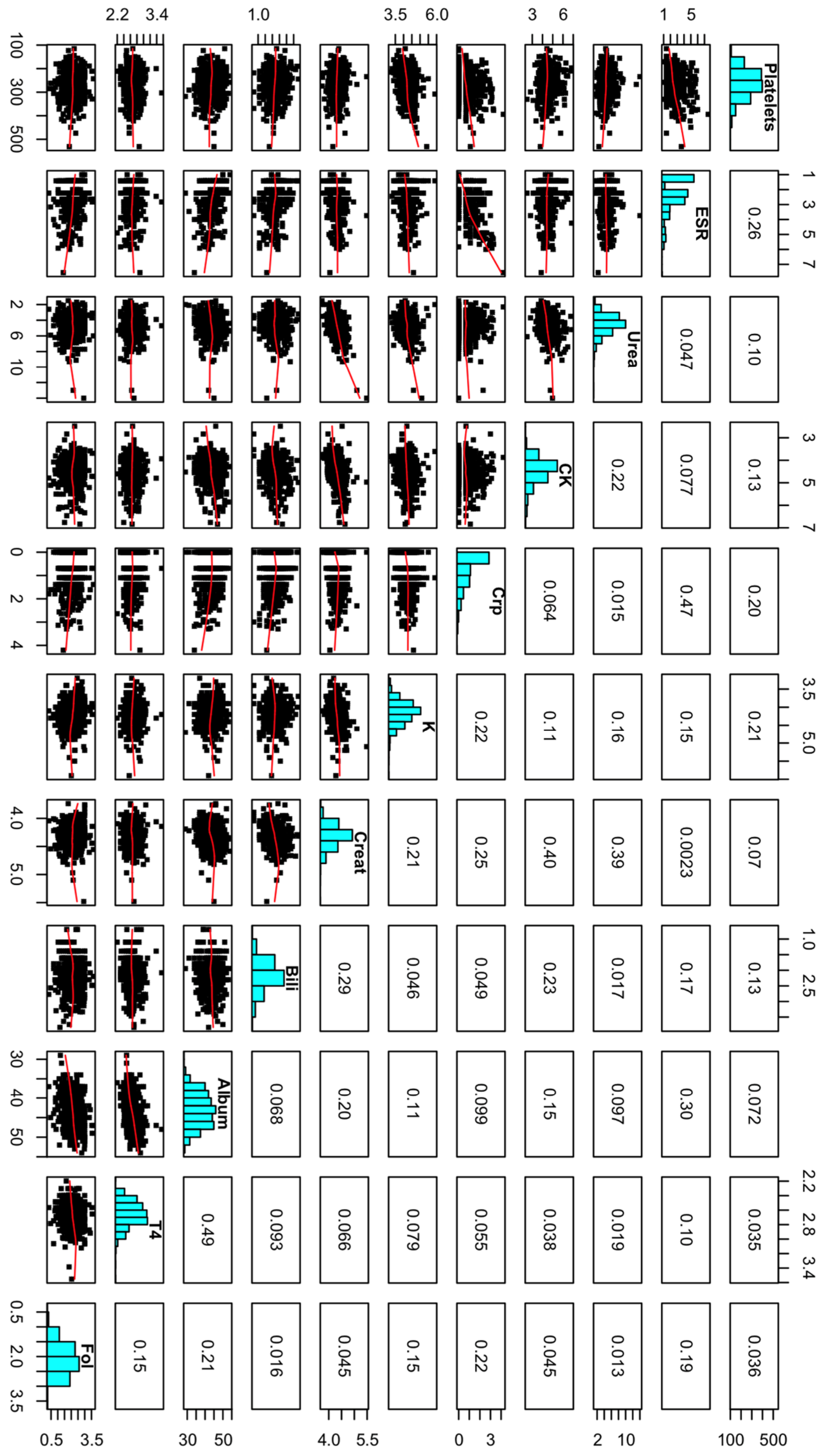

Figure S2. Laboratory tests across all samples.
